# Cross-reactivity of SARS-CoV-2– and influenza A–specific T cells in individuals exposed to SARS-CoV-2

**DOI:** 10.1172/jci.insight.158308

**Published:** 2022-09-22

**Authors:** Worarat Chaisawangwong, Hanzhi Wang, Theodore Kouo, Sebastian F. Salathe, Ariel Isser, Joan Glick Bieler, Maya L. Zhang, Natalie K. Livingston, Shuyi Li, Joseph J. Horowitz, Ron E. Samet, Israel Zyskind, Avi Z. Rosenberg, Jonathan P. Schneck

**Affiliations:** 1 Department of Pathology, School of Medicine,; 2 Department of Biomedical Engineering, Whiting School of Engineering,; 3 Department of Pediatrics, Division of Emergency Medicine,; 4 Department of Biomedical Engineering, School of Medicine, and; 5 Institute for Cell Engineering, School of Medicine, Johns Hopkins University, Baltimore, Maryland, USA.; 6 ExpressCare Urgent Care, Bel Air, Maryland, USA.; 7 Department of Anesthesiology, University of Maryland School of Medicine, Baltimore, Maryland, USA.; 8 Department of Pediatrics, NYU Langone Medical Center, New York, New York, USA; Maimonides Medical Center, New York, New York, USA.; 9 Institute for Nanobiotechnology and; 10 Department of Medicine, School of Medicine, Johns Hopkins University, Baltimore, Maryland, USA.

**Keywords:** COVID-19, Immunology, Adaptive immunity, T cells

## Abstract

Cross-reactive immunity between SARS-CoV-2 and other related coronaviruses has been well-documented, and it may play a role in preventing severe COVID-19. Epidemiological studies early in the pandemic showed a geographical association between high influenza vaccination rates and lower incidence of SARS-CoV-2 infection. We, therefore, analyzed whether exposure to influenza A virus (IAV) antigens could influence the T cell repertoire in response to SARS-CoV-2, indicating a heterologous immune response between these 2 unrelated viruses. Using artificial antigen-presenting cells (aAPCs) combined with real-time reverse-transcription PCR (RT-qPCR), we developed a sensitive assay to quickly screen for antigen-specific T cell responses and detected a significant correlation between responses to SARS-CoV-2 epitopes and IAV dominant epitope (M1_58–66_). Further analysis showed that some COVID-19 convalescent donors exhibited both T cell receptor (TCR) specificity and functional cytokine responses to multiple SARS-CoV-2 epitopes and M1_58–66_. Utilizing an aAPC-based stimulation/expansion assay, we detected cross-reactive T cells with specificity to SARS-CoV-2 and IAV. In addition, TCR sequencing of the cross-reactive and IAV-specific T cells revealed similarities between the TCR repertoires of the two populations. These results indicate that heterologous immunity shaped by our exposure to other unrelated endemic viruses may affect our immune response to novel viruses such as SARS-CoV-2.

## Graphical abstract

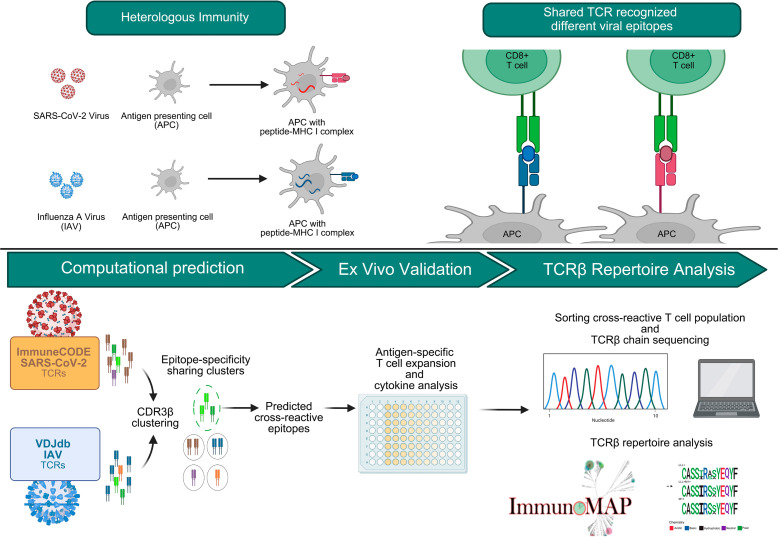

## Introduction

Since the emergence of the novel SARS-CoV-2 coronavirus in December 2019, the COVID-19 pandemic remains a global health crisis with over 6 million deaths worldwide (1). Disease severity ranges from asymptomatic or mild, influenza-like symptoms to acute respiratory distress syndrome, multiorgan failure, and death (2, 3). Yet, the mechanisms underlying this diversity in illness severity remain uncertain. One hypothesis is that heterologous immunity, the concept that a host may develop immunity to a certain pathogen after exposure to an unrelated pathogen, may modulate the immune response against SARS-CoV-2 in either a beneficial or detrimental manner (4–8). Heterologous immunity in T cells may occur via direct cross-reactivity of the T cell receptor (TCR) or indirectly via nonspecific activation of the T cell by circulating cytokines. In contrast to the “one-clonotype-one-specificity” paradigm established by clonal selection theory, studies have supported the hypothesis that the TCR possesses a tremendous amount of cross-reactivity to cover a wide range of possible foreign peptides (9–11). Much of this cross-reactivity is due to the ability of the TCR complementarity-determining region 3 (CDR3) to mold its conformation and fit various peptide-MHC complexes with varying degrees of affinity. Thus, heterologous immunity between two different viral antigens can even occur in the absence of shared peptide sequence homology (12–14). CDR3 is the most genetically diverse region of the TCR as it is derived from random genetic recombination of the V, D, and J gene segments for each individual (15). As a result, heterologous immunity between two antigens can vary significantly from person to person.

Recently, up to 81% of healthy, SARS-CoV-2–naive individuals have been shown to possess T cells reactive against epitopes belonging to endemic coronaviruses responsible for the “common cold” that are also reactive against SARS-CoV-2 epitopes (16, 17). The SARS-CoV-2 epitopes recognized by these T cells share similarities; these regard peptide sequence as well as biochemical and/or HLA-binding properties, with epitopes from the seasonal coronaviruses (HCoV-OC43, HCoV-229E, HCoV-NL63, HCoV-HKU1) (17). Epidemiological studies have also revealed an association between decreased incidence and severity of SARS-CoV-2 infection in regions with high influenza vaccination rates (18–21); however, there have been no studies to our knowledge to date examining the presence of heterologous immunity between SARS-CoV-2 and unrelated seasonal respiratory viruses such as influenza A virus (IAV). Heterologous immunity has also been shown in animal models to affect long-term immunity by disrupting the maintenance of memory T cell pools, reducing the diversity of the T cell repertoire, changing patterns of T cell immunodominance, and ultimately, contributing to the generation of viral escape variants (7). Because much of the population is exposed annually to IAV antigens either via infection or vaccination, we analyzed how this might influence the evolution of T cell immunity to SARS-CoV-2.

To investigate heterologous T cell immunity, traditional T cell assays, such as ELISpot, ELISA, and intracellular cytokine staining (ICS), have been used, but these are limited in sensitivity, given that T cells against any one specific antigen make up only a small percentage of the total T cell compartment (22). Based on these concerns, we developed what we believe to be a novel approach using artificial antigen-presenting cell (aAPC) technology (23) combined with real-time reverse-transcription PCR (RT-qPCR) of *IFNG* mRNA to screen cross-reactive T cell responses to SARS-CoV-2 and to IAV from whole blood. Herein, we identify a correlation between SARS-CoV-2– and IAV-specific CD8 T cell responses in individuals exposed to SARS-CoV-2. Using the aAPC platform to expand antigen-specific T cells, we found a population of cross-reactive T cells with specificity for both SARS-CoV-2– and IAV-derived epitopes in ex vivo expanded T cells. TCR-sequencing analysis of SARS-CoV-2– and IAV-specific CD8^+^ T cells isolated from convalescent donors confirmed a similar immune TCR repertoire in IAV-specific and cross-reactive T cells, implying a potential influence of IAV-specific CD8^+^ T cell precursors in the development of SARS-CoV-2 cross-reactive CD8^+^ T cell populations.

## Results

### Demographics of longitudinal study participants.

Peripheral blood samples (HLA-A: 02*01^+^) were obtained from 24 convalescent donors and 9 healthy donors prior to the FDA emergency use authorization of the Johnson & Johnson, Moderna, and Pfizer COVID-19 vaccines. Convalescent donors were enrolled as part of the Multi-institutional Study Analyzing Anti–CoV-2 Antibodies (MITZVA) (24); a subset of 12 donors (9 convalescent and 3 healthy donors) were followed longitudinally. SARS-CoV-2 infection was confirmed in convalescent donors either by a positive nasopharyngeal (NP) test or the presence of SARS-CoV-2 IgG. Healthy donors were defined as having a negative NP test and negative SARS-CoV-2 serology using EDI New Coronavirus COVID-19 IgM and IgG ELISA (Epitope Diagnostics Inc.). Whole blood samples were collected early in the pandemic at the time of study enrollment and 7 weeks later. Demographics of all donors and those who were followed longitudinally whose peripheral blood samples were used for this study are shown in Table 1 and Table 2, respectively.

### Development of the SARS-CoV-2 aAPC IFNG RT-qPCR assay.

**Figure 1:**
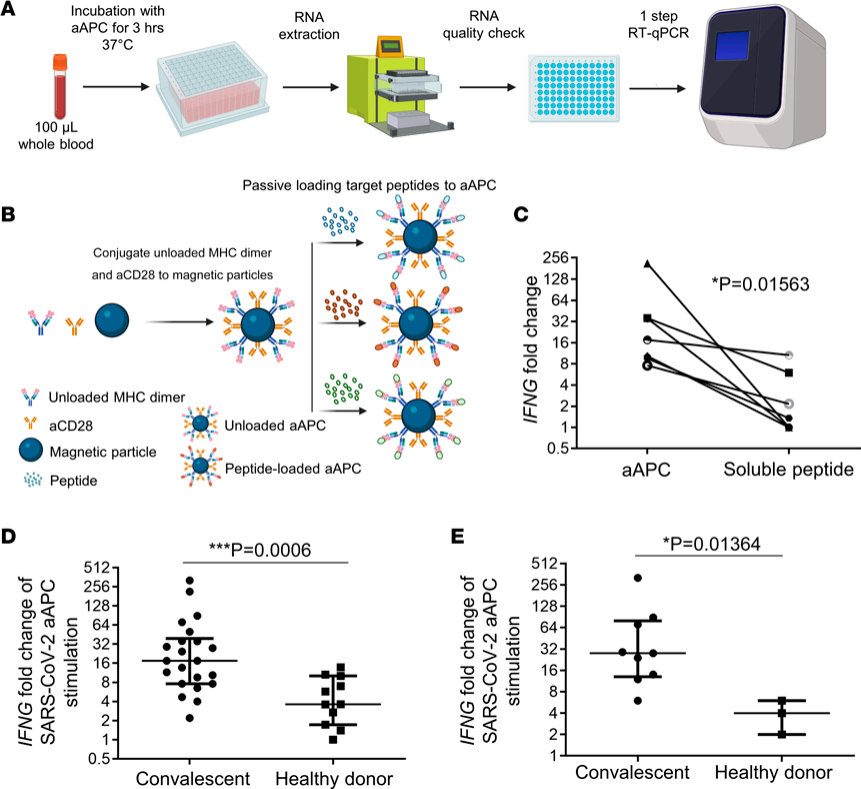
Antigen-specific T cell responses using a rapid aAPC-based assay. (**A**) Schematic view of the experimental workflow for determining *IFNG* mRNA fold change using aAPC and 1-step RT-qPCR. (**B**) Schematic illustration of adaptive aAPC process. Unloaded MHC dimer and αCD28 are first conjugated to magnetic particles. Peptides of interest are then passively loaded onto the empty MHC by coincubation to generate peptide-specific aAPC. (**C**) *IFNG* fold change after stimulation for 3 hours with either aAPC or soluble peptide in matched samples. Significance was determined by 2-tailed Wilcoxon matched-pairs signed rank test (*n* = 7). (**D**) Comparison of median *IFNG* fold change above that of unstimulated samples in convalescent (*n* = 24) versus uninfected individuals (*n* = 11). Median fold change above that of matched unstimulated samples in *IFNG* mRNA after stimulation with pooled 6 SARS-CV2 peptide–aAPCs (shown in Supplemental Table 1). Significance was determined by 2-tailed Mann-Whitney *U* test, with threshold of *P* ≤ 0.05 (**E**) Comparison of median *IFNG* fold change above that of unstimulated samples in convalescent (*n* = 9) versus uninfected individuals (*n* = 3) followed longitudinally. Median fold change that of above matched unstimulated samples in *IFNG* mRNA after stimulation with batched peptide-aAPC. Significance was determined by 2-tailed Mann-Whitney *U* test, with threshold of *P* ≤ 0.05.

To screen samples in a high-throughput manner, we first developed a rapid assay to analyze antigen-specific CD8^+^ T cells using aAPC technology (23) combined with RT-qPCR for *IFNG*. This assay enables the identification of patients with memory CD8^+^ T cell responses (25) against previously defined immunodominant epitopes of SARS-CoV-2 (26) (Figure 1A; peptides are listed in Supplemental Table 1; supplemental material available online with this article; https://doi.org/10.1172/jci.insight.158308DS1). The predesigned primers are indicated in Supplemental Table 3. Briefly, aAPCs are paramagnetic iron particles conjugated with unloaded HLA-A: 02*01 dimer (signal 1) and αCD28 IgG (signal 2). Epitopes of interest are loaded onto the aAPC as described previously (23) (Figure 1B; peptides are listed in Supplemental Table 1). One hundred microliters of whole blood were then incubated for 3 hours at 37°C with either pooled SARS-CoV-2 peptide–loaded aAPCs or M1-loaded aAPCs. Fold change above that of unstimulated controls was calculated using the 2^–ΔΔCt^ as the comparative Ct method. To assess the performance of the multiplex 1-step RT-qPCR assay, we first calculated the amplification efficiency for all the primer pairs analyzed in this study. RT-qPCR primer efficiency was then obtained from the slopes of their corresponding standard curves. Primer efficiencies ranged from 95% to 110%, with the optimal efficiency range being 80%–110%. The standard curves showed high linearity with correlation coefficients (R^2^) between 0.9949 and 0.9998 (Supplemental Figure 1). For quality control purposes, this assay must be performed on fresh whole blood to avoid signal degradation and artifact resulting from freezing/thawing of PBMCs.

Compared with traditional soluble antigen-based stimulation, which relies on endogenous APCs, our aAPC-based approach was more sensitive to detecting T cell responses after only a 3-hour incubation. We found significant differences in *IFNG* fold change, with a median of 17.52 (IQR, 9.56–35.84), when using aAPCs compared with a median of 1.35 (IQR, 1–5.98) when using soluble peptide alone in matched individuals (*P* = 0.0156) (Figure 1C and Supplemental Table 1).

We analyzed the median *IFNG* fold change in all 33 donors. Stimulation of whole blood samples with SARS-CoV-2 peptide–loaded aAPCs demonstrated a significantly higher *IFNG* response (median = 17; IQR, 8–39) in convalescent individuals compared with those in the healthy donor group (median = 3; IQR, 1–3) (Figure 1D). Similarly, analysis of *IFNG* responses in our longitudinal cohort demonstrated significantly higher median *IFNG* fold changes in the convalescent group (median = 28; IQR, 13–80) compared with those in the healthy donor group (median = 4; IQR, 2–6) (Figure 1E). Together these results demonstrate that an aAPC-based qPCR assay developed to evaluate antigen-specific T cell responses is more sensitive and faster than conventional soluble peptide–based assays.

### Correlation between SARS-CoV-2 and IAV CD8^+^ T cell responses.

**Figure 2:**
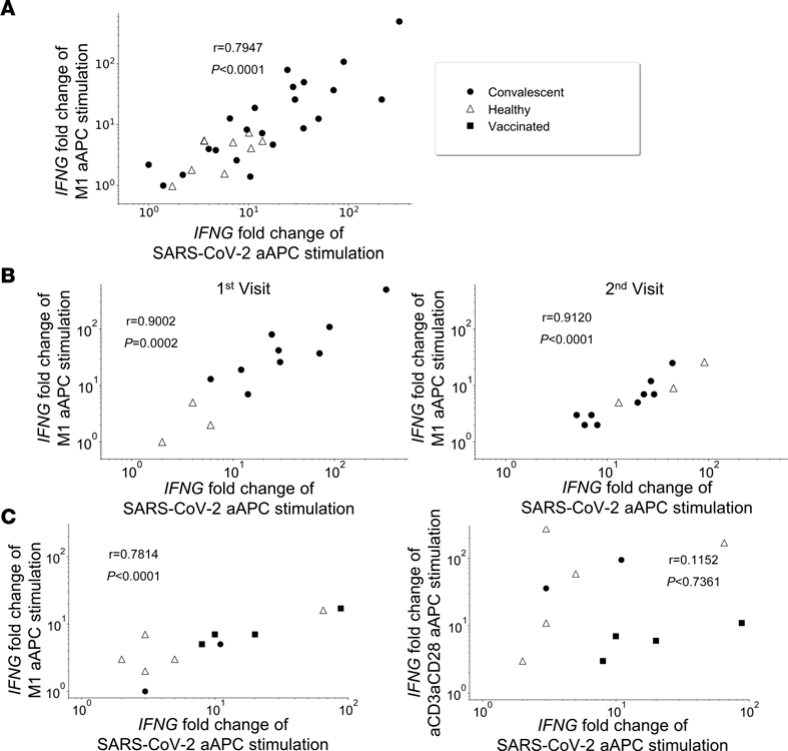
Correlation between CD8 T cell responses to SARS-CoV-2 and M1 but not to nonspecific activation with αCD3/αCD28. (**A**) Correlation between SARS-CoV-2 and M1 in all 33 donors. Scatter plots comparing *IFNG* fold change after stimulation with pooled 6 SARS-CoV-2 aAPCs and M1 aAPCs. (**B**) Correlation between SARS-CoV-2 and M1 persisted longitudinally in 12 donors. Scatter plots comparing *IFNG* fold change after stimulation with SARS-CoV-2 aAPCs and M1 aAPCs at the first visit 7 days after exposure to SARS-CoV-2 (left) and the second visit 7 weeks later (right). (**C**) Scatter plots comparing *IFNG* fold change after stimulation with SARS-CoV-2 aAPCs and M1 aAPCs (left) as well as αCD3/αCD28 aAPCs (right) (*n* = 5, healthy donors [triangles]; *n* = 2, convalescent individuals [circles]; *n* = 4, vaccinated individuals [black squares]). Two-sided Spearman’s rank correlation was used to test for significance. Calculated *P* and *r* values (correlation coefficient) are indicated.

To test our hypothesis that heterologous immunity exists between SARS-CoV-2– and IAV-specific memory CD8^+^ T cell responses, we stimulated whole blood samples from convalescent patients with COVID-19 with aAPCs loaded with a pool of 6 SARS-CoV-2 T epitopes that had previously been confirmed using unbiased ex vivo screens (26). Then, we compared the resulting *IFNG* response to stimulation with aAPCs loaded with the IAV HLA-A*02:01 immunodominant peptide, M1_58–66_. We found a correlation between SARS-CoV-2– and IAV-specific CD8^+^ T cell reactivity (*r =* 0.7947; *P <* 0.0001) (Figure 2A) in 33 donors. We found a similar correlation (*r* = 0.9002; *P =* 0.0002) in the longitudinal cohort (Figure 2B). This correlation persisted for at least 7 weeks, as was seen after the second visit (*r =* 0.9120; *P <* 0.0001) (Figure 2B). Thus, we observed a statistically significant correlation between *IFNG* mRNA expression in response to aAPC stimulation with SARS-CoV-2 peptides and IAV M1 at both visits.

We next asked whether this correlation was specific to SARS-CoV-2 and M1 or rather overall increased T cell responsiveness in COVID-19 convalescent donors as compared with healthy donors. To address this, we examined whether a similar correlation was seen between samples stimulated with SARS-CoV-2 and samples stimulated nonspecifically with αCD3/αCD28 particles. Due to the need to run this assay on fresh whole blood, we tested this relationship in a subset of 11 donors, from whom we obtained fresh samples. We found that a significant correlation was only detected with M1 (*r* = 0.7814; *P* = 0.0063) but not with nonspecific activation with αCD3/αCD28 microparticles (*r* = 0.1152; *P* = 0.7361) (Figure 2C). This result suggests that the mechanism behind the previously observed correlation between SARS-CoV-2 and IAV T cell response (Figure 2) is due to antigen recognition at the TCR level rather than the underlying activation state of the T cells.

### Cross-reactivity of SARS-CoV-2– and M1-specific T cells.

We then investigated whether we could expand the cross-reactive CD8^+^ T cell population with peptide-loaded aAPCs for downstream analysis. To overcome the limited availability of the samples needed for a broader screen, we sought to develop a novel method to computationally infer the SARS-CoV-2 epitopes with the highest likelihood of being recognized by M1-specific T cells. For this analysis, we cross-referenced two publicly available TCR binding data sets, VDJdb (27) and ImmuneCODE (28), as TCRβ chains with similar CDR3β sequences may share the same antigenic specificities (29–31).

**Figure 3:**
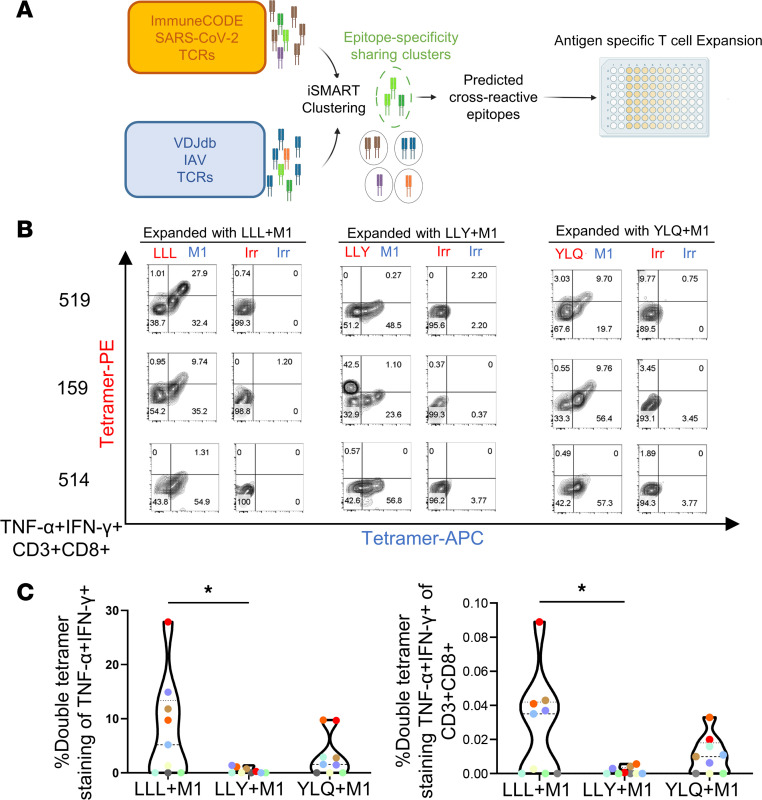
Functional cross reactivity between CD8 T cell responses to SARS-CoV-2 and M1. (**A**) Overview of cross-reference search between the ImmuneCODE Adaptive-MIRA database and the VDJdb public data set, followed by a 14-day expansion of PBMCs with aAPCs. (**B**) Representative flow plots of CD3^+^CD8^+^TNF-α^+^IFN-γ^+^ T cell populations, following a 14-day expansion of PBMCs from COVID-19 convalescent individuals with mixed 3 pairs: (a) LLL and M1, (b) LLY and M1, and (c) YLQ and M1 versus irrelevant (Irr). Flow plots illustrate dual-tetramer staining of the cytokine-producing CD8^+^ T cells with tetramers labeled with either PE (red) or APC (blue). (**C**) Double-tetramer staining of 14-day expanded PBMCs. The percentage of polyfunctional CD8^+^ T cells that was double tetramer positive (left), and the percentage of the total CD8^+^ T cell population that was both polyfunctional and double tetramer positive (right). Two-sided 1-way ANOVA with Tukey’s multiple comparison was used to test for significance. *P < 0.05.

CDR3β sequences with known specificities to immunodominant SARS-CoV-2 epitopes used in our qPCR screen (Supplemental Table 1) were extracted from ImmuneCODE (4,425 entries; Figure 3A), along with CDR3β sequences specific for M1 that were extracted from VDJdb (3,407 entries). We then applied the TCR clustering algorithm immuno-Similarity Measurement by Aligning Receptors of T cells (iSMART) (32) to identify TCR clusters with potentially shared specificities based on CDR3β similarity. Among all 6 immunodominant SARS-CoV-2 epitopes, LLLDRLNQL (LLL), LLYDANYFL (LLY), and YLQPRTFLL (YLQ) had the highest amount of CDR3b sequences similar to M1-specific TCR entries (Supplemental Table 2). Notably, 6 M1-specific TCRs mapped exactly with the same CDR3β sequences as LLL-specific TCRs.

To validate our approach, the LLL epitope was selected to expand SARS-CoV-2 and IAV cross-reactive T cells. This was done using our aAPC system to expand PBMCs from 4 convalescent donors ex vivo with aAPCs loaded with a 1:1 ratio of the LLL and M1 peptides for 14 days. T cells specific for the LLL epitope were confirmed by double-tetramer staining using LLL-PE and LLL-APC (data not shown) and were found to be in the range of 0.014%–0.14% of CD8^+^ T cells for 3 of the 4 donors. Costaining with LLL and M1 tetramer was also performed on day 14. Following expansion with mixed LLL- and M1-loaded aAPCs, we observed LLL-specific T cells in the range of 0.018%–0.16%, which corresponds well to double-tetramer staining with LLL-PE and LLL-APC (Supplemental Figure 2B). Moreover, we were able to identify a population of CD8^+^ T cells from 3 of 4 convalescent donors that costained with both LLL and M1 tetramers. These results show that a subset of LLL-specific CD8^+^ T cells binds specifically to both LLL and M1 epitopes.

Additionally, to broaden the analysis, we studied 9 donors with 3 SARS-CoV-2 epitopes (LLL, LLY, and YLQ) to test for the presence and functionality of TCR cross-reactivity between SARS-CoV-2 and IAV. These studies were done in combination with ICS to demonstrate functional cross-reactivity. We again used our aAPC system to expand PBMCs from the 9 convalescent donors ex vivo with different combinations of aAPCs loaded with either SARS-CoV-2 epitope or M1 (LLL and M1, LLY and M1, and YLQ and M1) for 14 days. Expanded cells were restimulated with matched SARS-CoV-2 peptide–loaded aAPCs and analyzed for cytokine production. Cytokine-producing cells were then assessed for TCR cross-reactivity by their ability to costain with both the SARS-CoV-2 peptide tetramer of interest and the M1 tetramer (see Supplemental Figure 3 for gating strategy). Nonspecific binding was determined using a tetramer loaded with an irrelevant HLA-A*02:01 binding peptide.

The dual TNF-α/IFN-γ–producing cells were stained with matched SARS-CoV-2 epitope-specific tetramers and M1 tetramers. Six of 9 donors showed costaining with LLL and M1 tetramers in the LLL/M1 aAPC-expanded group, and 7 of 9 donors showed costaining with YLQ and M1 tetramers in the YLQ/M1 aAPC-expanded group. By comparison, only trace amounts of cytokine-producing cells stained with the irrelevant tetramers, serving as a negative control and, thus, demonstrating an antigen-specific response to the SARS-CoV-2 LLL/YLQ and IAV M1 epitopes (see Figure 3B for representative examples and Supplemental Figure 4 for other donor populations). Costaining with LLL and M1 tetramer was found in 27.9% and 9.74% of cytokine-producing cells from donors 519 and 159, respectively, while costaining of YLQ and M1 was found in 9.70% and 9.76% of cytokine-producing cells, respectively (Figure 3B). We did not observe costaining of LLY and M1 in the expanded polyfunctional LLY/M1 group in either donor (Figure 3B and Supplemental Figure 4). Nevertheless, donor 159 showed distinct LLY^+^ populations (42.5%) and M1^+^ populations (23.6%) (Figure 3B, middle), showing that this donor had SARS-CoV-2 LLY–T cell–specific responses that simply did not cross-react with their M1 response. In contrast, while donor 514 showed a high percentage of M1-specific T cells (54.9%–57.3%), we did not observe costaining with YLQ or LLY tetramer and found that only 1.3% of cytokine-producing cells costained with LLL and M1 tetramer (Figure 3B). Thus, we observed varying extents of cross-reactivity in each individual, as would be expected given the diversity of the human population.

The LLL^+^M1^+^-specific T cells were consistently present at higher frequencies compared with other epitopes tested for multiple individuals at the median of 5% (±9.391%) of polyfunctional T cells (TNF-α^+^IFN-γ^+^), which accounted for 0.035% (±0.03%) of all CD8^+^ T cells (*P* < 0.05) (Figure 3C). The YLQ^+^M1^+^-specific T cells accounted for 1.57% (±3.904%) of polyfunctional T cells and 0.01% (±0.01%) of all CD8^+^ T cells (Figure 3C) but were not statistically different from the LLY^+^M1^+^-specific T cells. Taken together, these data demonstrate the existence of varying degrees of heterologous immunity between SARS-CoV-2 and IAV epitopes, with LLL and M1 cross-reactive CD8^+^ T cells being the most prevalent of those tested.

### SARS-CoV-2 cross-reactive TCRs share similarity with IAV-reactive TCRs.

**Figure 4:**
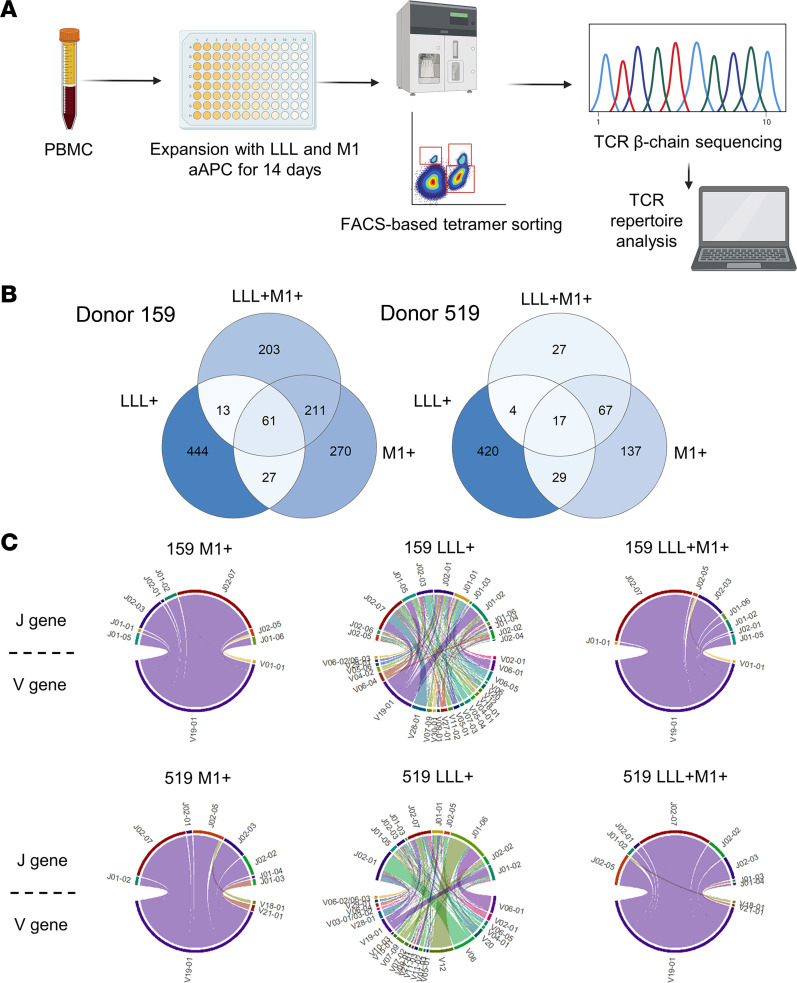
Expansion and TCR-sequencing analysis of T cell populations with different specificities. (**A**) Overview of the analysis workflow. PBMCs from 2 donors (donors 159 and 519) were expanded with mixed aAPCs loaded with LLL and M1 epitopes for 14 days. Tetramer staining and FACS-based sorting was performed to select LLL^+^, LLL^+^M1^+^, and M1^+^ cells, which underwent TCR sequencing and analysis. (**B**) Venn diagrams showing that TCR overlaps with shared CDR3β sequences among samples. (**C**) Chord diagrams showing V and J gene usage in each sample. The top half of the circles shows J genes, and the bottom shows V genes. Each link denotes clones with the same VJ gene usage. The width of links is scaled to the productive frequency of the clones.

Having identified cross-reactive CD8^+^ T cells, we studied the TCR repertoire of LLL-M1 cross-reactive CD8^+^ T cells. The composition of cross-reactive T cells was analyzed by FACS sorting on PBMCs from 2 convalescent donors following a 14-day expansion. Three CD8^+^ T cell populations were sorted and selected for TCR sequencing, LLL^+^, LLL^+^M1^+^, and M1^+^ (Figure 4A). From these, we derived 2,796 unique TCR entries. Because TCRs with similar CDR3β sequences may identify the same epitope (33), we first compared the sorted repertoire with online databases (VDJdb and ImmuneCODE) using iSMART. While 84.5% of M1^+^ T cells from donor 159 and 88.1% of M1^+^ T cells from donor 519 were clustered with VDJdb M1-specific TCRs, only 5.5% of LLL^+^ T cells from donor 159 and 5.7% of LLL^+^ T cells from donor 519 were clustered with ImmuneCODE LLL-specific TCRs, indicating a heterogenous LLL-specific response encompassing a large repertoire of TCRs that had not been recorded in the database. Of note, 5.52% of LLL^+^M1^+^ T cells from donor 159 and 4.19% of LLL^+^M1^+^ T cells from donor 519 were clustered with our previously predicted potentially cross-reactive TCRs.

To elucidate the relationship between TCR repertoires, we analyzed CDR3β sharing among different T cell populations. We found high CDR3β overlap between the M1^+^ population and the cross-reactive LLL^+^M1^+^ population in both donors (211 clones in donor 159; 67 clones in donor 519); whereas most CDR3βs in LLL^+^ populations were not shared with the other 2 populations: 444 of 545 LLL^+^ clones were unique in donor 159, and 420 of 470 LLL^+^ clones were unique in donor 519 (Figure 4B). This suggested more common precursors for M1^+^ and cross-reactive CD8^+^ T cells but less for LLL^+^ T cells. Consistent with this congruence in M1^+^ and LLL^+^M1^+^ clones, we discovered a skewed usage of the TCRBV19-01 gene in both M1^+^ and LLL^+^M1^+^ populations from both donors, compared with a published CD8^+^ TCR data set (34) of 4 healthy adult donors (Figure 4C and Supplemental Figure 5). Strikingly, more than 90% of clones in M1^+^ and LLL^+^M1^+^ T cells had TCRBV19-01 usage, further underscoring a relationship between M1^+^ and LLL^+^M1^+^ cross-reactive T cells. This analysis indicates that the CD8^+^ LLL^+^M1^+^ cross-reactive T cell repertoire was potentially influenced by, or partially originated from, preexisting M1-specific immunity.

### ImmunoMap visualization of immune repertoire of cross-reactive T cells.

**Figure 5:**
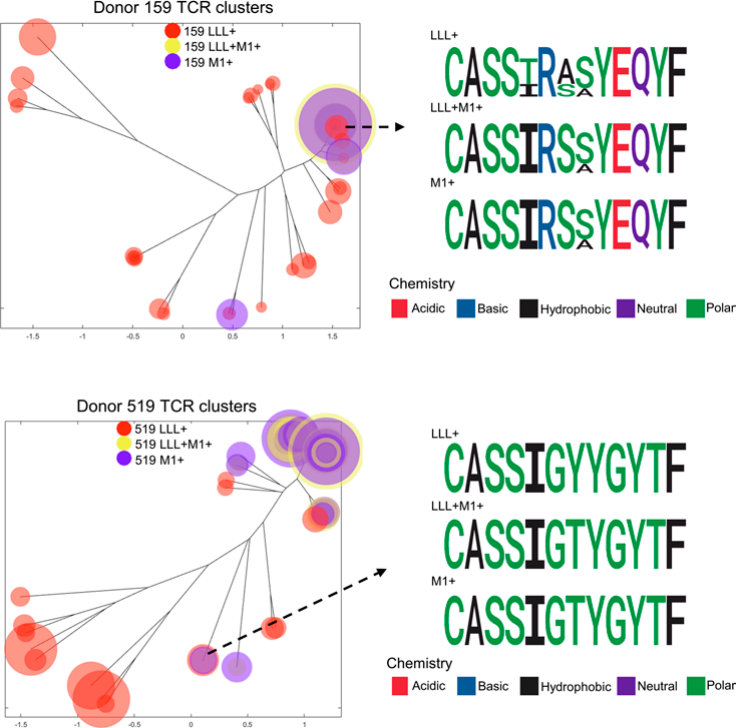
ImmunoMap clustering of sorted T cell samples from 2 donors. Each circle represents a cluster, whose size is proportional to the total frequency of clones within the cluster. Clusters with common motifs in all 3 samples for each donor were selected and visualized by logo plot; different colors denote varying amino acid chemistry.

To explore the similarities of CDR3β sequences of enriched clonotypes, we used ImmunoMap (35) to cluster and visualize clones (Figure 5). Apart from identifying shared CDR3β sequences, ImmunoMap grouped CDR3β epitopes with high sequence similarity into clusters, depicted by circles with sizes that are proportional to clonal frequency. ImmunoMap analysis showed a high similarity between M1^+^ and LLL^+^M1^+^ populations, as shown by the vicinity of the clusters (Figure 5). Among the clusters, the enriched motif “CASS%R%%YEQYF” was present in all populations of one donor (donor 159), while “CASSIG%YGYTF” was present in all populations of the second donor (donor 519), where “%” stands for varying amino acids. Collectively, these data indicate a remarkable clonal overlap between the LLL^+^M1^+^ cross-reactive T cells and M1^+^-specific T cells, hinting at a common precursor T cell population.

## Discussion

We developed an assay for SARS-CoV-2–specific T cell responses that is faster and more sensitive than traditional functional T cell assays. Using this approach, we identified a correlation between SARS-CoV-2– and IAV-specific CD8^+^ T cell responses, suggesting the presence of preexisting heterologous immunity. To our knowledge, this is the first identification of the potential for heterologous immunity between SARS-CoV-2 and IAV, 2 distinct respiratory viruses with minimal overlap in peptide sequence homology.

Using aAPC technology coupled with RT-qPCR, we developed an assay to evaluate antigen-specific T cell responses in patients exposed to SARS-CoV-2. This approach has two major advantages over traditional soluble peptide–based approaches. First, our aAPC approach does not rely on endogenous APCs, which can be highly variable in quantity and quality from patient to patient. Indeed, endogenous APCs are dysfunctional in some patients with COVID-19 (36, 37). Second, we demonstrate that this approach is more sensitive and faster while using only a small amount (100 μL) of unprocessed whole blood. This assay can be easily scaled and automated for high-throughput processing. At this proof-of-principle stage, the technology is limited to HLA-A*02:01 individuals; however, more HLA class I or HLA class II alleles can be adapted and applied for broader studies.

We showed a correlation between T cell responses to SARS-CoV-2 and the immunodominant epitope, M1_58–66_ (GILGFVFTL), of IAV. These findings demonstrate that a fraction of M1-specific T cells induced by IAV infection are potentially cross-reactive with antigens from SARS-CoV-2 and support the hypothesis that preexisting immunity to IAV may influence the cellular immune response to SARS-CoV-2 antigens. We further identified potential cross-reactive T cell clones and their epitopes by cross-referencing two publicly available TCR binding data sets, VDJb for IAV M1 (27) and ImmuneCODE for SARS-CoV-2 (28). Rather than searching for shared epitope sequence homology, we utilized an unbiased approach by first evaluating for similar TCR CDR3β sequences responding to both viruses. This approach has the potential to uncover many more cross-reactive epitopes, as it is not restricted solely to shared peptide sequence homology but also takes into consideration other factors important for TCR binding, such as CDR3 loop flexibility, different peptide-binding registers/angles, and residue-focused TCR engagement, which allows amino acid substitutions at other locations. Our analyses revealed multiple shared TCR CDR3β sequence candidates for cross-reactive T cell clones in addition to the 6 immunodominant SARS-CoV-2 epitopes used in our qPCR screen.

To select for cross-reactive T cells from each donor, we stimulated cells with (a) LLL and M1, (b) LLY and M1, and (c) YLQ and M1 peptide–loaded aAPCs for 14 days. Cross-reactive T cells in this population were confirmed by ICS and tetramer costaining studies. Thus, this potentially novel approach, which can be used with limited clinical samples, revealed a cross-reactive CD8^+^ T cell population.

To demonstrate a proof of concept supporting TCR sequence homology, rather than strict peptide homology to identify cross-reactive T cell populations, we analyzed both spike (YLQ) and nonspike protein (LLL and LLY) epitopes. Utilizing the CDR3β information from publicly available TCR databases, we screened for potentially cross-reactive TCR clones and computationally inferred cross-reactive epitopes. We then experimentally documented the cross-reactive TCR in our donor population. However, because SARS-CoV-2–specific T cell responses are highly heterogeneous among different individuals, TCR records for different SARS-CoV-2 peptides are still limited. Thus, a more thorough and comprehensive database is needed to increase the sensitivity and accuracy of our computational prediction approach.

In this study, we focused on the cross-reactivity of selected SARS-CoV-2 CD8^+^ T cells with T cells specific for M1, an epitope derived from an unrelated respiratory virus, IAV. Other groups have shown the prevalence of cross-reactive memory CD4^+^ T cells between SARS-CoV-2 and other-related endemic coronaviruses in the range of 20%–50% (38–40). By applying our technique to study cross-reactivity in CD4^+^ T cells, we could potentially unravel more cross-reactive epitopes and more heterologous cross-reactive T cell populations. Cross-reactive T cells should not be presumed to provide protective immunity but can still significantly alter the T cell repertoire and, thus, potentially provide guidance for further vaccine development and treatment. Additional research will be critical to fully understand the nature of these cross-reactive T cells and their correlation with clinical outcomes.

Our findings raise the possibility that the T cell repertoire in response to new, emerging viruses such as SARS-CoV-2 is shaped by the accumulation of our prior exposures to circulating, endemic viruses via heterologous immunity. While the presence of a pool of cross-reactive memory T cells already primed to quickly respond to a new antigen may provide an initial survival benefit to the host by mitigating viral replication, heterologous immunity may also be detrimental in the long run by narrowing TCR diversity, likely in the older population, to a broad array of viral epitopes and, thus, promote the development of viral escape variants. It is also important to note that, because the TCR CDR3 region, which enables the TCR to be cross-reactive across multiple different epitopes, is the most genetically variable region from person to person, the repertoire of cross-reactive T cells will vary from one person to the next. This concept is more broadly known as an individual’s “private repertoire” and may be key to understanding determinants of disease severity in COVID-19 as well as which hosts are more prone to generating viral escape variants. Though we only focused on cross-reactivity to IAV in this report, similar concepts are at play with regard to other endemic respiratory viruses, such as a respiratory syncytial virus, parainfluenza, and human metapneumovirus, etc. Future directions can focus on understanding how exposures to common respiratory viruses and vaccines may affect heterologous immunity to unrelated viruses. We believe that these findings have novel implications for the understanding of protective immunity to seasonal respiratory viruses as well as factors potentially influencing the design of universal vaccines.

## Methods

### Blood sample processing.

Eight milliliters of peripheral blood were obtained by venipuncture using heparin collection tubes. 300–600 μL peripheral blood was used for the aAPC-based qPCR assay. PBMCs were isolated via Ficoll-Paque (GE Healthcare Biosciences) according to the manufacturer’s instructions. Briefly, whole blood was centrifuged for 10 minutes at 757*g* to separate plasma and isolated by density-gradient centrifugation using Ficoll-Paque. Isolated PBMCs were cryopreserved in FBS containing 10% DMSO (MilliporeSigma) and stored in liquid nitrogen until further use.

### aAPC SARS-CoV-2 peptide stimulation.

Human antigen-specific CD8^+^ T cell activation was performed with in-house generated MHC-peptide/anti-human CD28 monoclonal antibody–coated (Bio X cell, clone 9.3) Dynabeads (4.5 μm, Invitrogen) (Figure 1B). The 6 HLA-A:02*01 immunodominant epitopes of SARS-CoV-2 (Supplemental Table 1) and IAV M1 epitope (GILGFVFTL) were passively loaded onto aAPCs, as previously described (23). One hundred microliters of fresh whole blood were diluted in 400 μL PBS and then incubated with peptide-loaded aAPCs. After stimulation, whole blood was lysed with erythrocyte lysis buffer (Qiagen), and cells were pelleted by centrifugation. RNA was isolated following the PureLink Pro 96 RNA extraction protocol and then stored at –80°C until use.

### RT-qPCR.

RNA was thawed on ice. Concentration and purity were determined by nanodrop at wavelength A260/280 (OD260/OD280 ratio). The 1-step RT-PCR reaction was performed using TaqMan Fast Virus 1-Step Master Mix (Thermo Fisher Scientific). Quantitative real-time PCR was performed using QuantStudio 6 (Applied Biosystems) and QuantStudio Real-time PCR software, version 1.1 (Applied Biosystems). All primers were predesigned by Thermo Fisher Scientific and are shown in Supplemental Table 3. All reactions were run in duplicates in 96-well plates (Thermo Fisher Scientific). For all primer pairs, standard curves were generated based on a 2-fold dilution series of a pool of different RNA from samples analyzed in this work. Amplification efficiencies and correlation coefficients for each primer pair were calculated from the slopes of the standard curves. *IFNG* Ct values were normalized to *CD8* and *HPRT1* mRNA for all assay conditions, and final data were reported as the relative *IFNG* fold change above that of unstimulated controls using the comparative Ct (ΔΔCt) method.

### TCR public database selection.

For putative cross-reactive epitope identification, two publicly available data sets were used in this study: the VDJdb (27) and ImmuneCODE database (28). For M1, the immunodominant epitope of influenza A, the antigen-binding data were downloaded from the VDJdb database (27) with the following filter options: antigen source species, influenza A; epitope-sequence or pattern, “GILGFVFTL.” To extract the TCRβ sequence for cross-referencing with ImmuneCODE, we further selected data satisfying the following requirements: gene, TRB; species, Homo sapiens. Duplicate records with the same CDR3 sequence and V gene and J gene arrangements were removed. Note that records with the same CDR3β sequence but different V/J gene rearrangements were not considered duplicates. ImmuneCODE data containing CDR3β sequences binding to SARS-CoV-2 epitopes were downloaded from the website with the filename of “ImmuneCODE-MIRA-Release002.1.” Specifically, records in “peptide-detail-ci.tsv” and “peptide-detail-cii.tsv” were combined for the cross-reference. Duplicate records with the same TCR sequence, V gene, J gene, experiment ID, start and end index in genome, and ORF coverage were removed. Healthy donor CD8^+^ T cell–sequencing data were downloaded from ImmuneACCESS (Adaptive Biotechnologies) (34).

### TCR cross-reference of different data sets.

Among filtered records from public TCR databases, CDR3β binding to our SARS-CoV-2 epitope pools (YLQ, LLL, KLWAQCVQL, ALWEIQQVV, YLFDESGEFKL, LLY) was selected and reported. CDR3β records from the VDJdb and ImmuneCODE database were compared and clustered using iSMART.

### Data analysis of TCR repertoire and clustering with iSMART and ImmunoMap.

TCR sequencing data were downloaded from ImmuneACCESS (Adaptive Biotechnologies) and processed with R. Duplicate entries with identical CDR3β sequences and V(D)J genes were combined. Venn diagrams and chord diagrams were generated in R. For visualization purposes, only TCR entries with more than 0.5% productive frequency were included for the chord diagrams. iSMART (32) was downloaded from Github (41) and implemented with default parameters after adaptation from Python 2 to Python 3. The ImmunoMap (35) package was downloaded from Github (42) and implemented via MATLAB R2020A. Suggested parameters from the authors were used and listed as follows: homology threshold, 0.1; cluster frequency threshold, 1; PAM10 matrix; gap penalty, 30. After homologous clusters were summarized in ImmunoMap, R was used to generate the sequence logo plot. The iSMART and ImmunoMap source codes used in this study can be found on Github (41, 42).

### Flow cytometric sorting.

PBMCs from 2 SARS-CoV-2 convalescent donors were expanded with LLL and M1 aAPCs for 14 days; stained with CD3, CD8, L/D, and LLL and M1 tetramers; and sorted on a MoFlo sorter (Beckman Coulter) Three fractions were collected: (a) double staining with LLL^+^ and M1^+^ tetramer; (b) LLL^+^ tetramer only; and (c) M1^+^ tetramer only.

### High-throughput TCR sequencing.

TCRβ CDR3 regions were amplified and sequenced using the protocol from Adaptive Biotechnologies (43). In brief, the method applies a multiplex PCR system to amplify all possible rearranged TCRβ CDR3 sequences from cDNA samples; results were compatible with those from the Illumina HiSeq cluster station solid-phase PCR system. The raw HiSeq sequence data were preprocessed to remove errors and compress the data. Analysis of TCRβ sequences was conducted with the Adaptive Biotechnologies TCR ImmunoSEQ assay. The TCR CDR3 region nomenclature was defined according to the International Immunogenetics collaboration (43), beginning with the second conserved cysteine encoded by the 3′ portion of the Vβ gene segment and ending with the conserved phenylalanine encoded by the 5′ portion of the Jβ gene segment. The number of nucleotides between these codons determines the length and, therefore, the frame of the CDR3 region. To identify which V, D, and J segments contributed to each TCR CDR3 sequence a standard algorithm was used.

### Ex vivo antigen-specific expansion and ICS.

Freshly thawed PBMCs were cultured overnight at 37°C with 5% CO_2_ in supplemented RPMI with 10% Human-Ab serum (GeminiBio). The rested cells were washed, and2million PBMCs were stimulated using aAPC described previously (23) in supplemented RPMI with 10% Human-Ab serum plus mixed cytokines (4 ng/mL IL-2, 0.3 ng/mL IL-4, 0.4 ng/mL IL-6, 0.2 ng/mL IL-1b, and 1 ng/mL IFN-γ). On day 7, 2 million thawed PBMCs were replated with aAPCs. The cells were fed on days 3, 5, 10, and 12. On day 14, cells were stimulated with aAPCs and Golgi plug for 6 hours and then stained with tetramers (LLL and YLQ, NIH Tetramer Core Facility; M1 and MART1 from MBL; and LLY from Tetramer Shop) at room temperature for 30 minutes before staining with the LIVE/DEAD fixable aqua dead cell stain kit (Invitrogen) and anti-CD3 (clone SK7) and anti-CD8 (clone SKI) antibodies at 4°C for 30 minutes. Cells were subsequently fixed and permeabilized using the Cytofix/Cytoperm kit (BD Biosciences — Pharmingen) and stained with anti–IFN-γ (clone 4S.B3) and anti–TNF-α (clone MAb11) antibodies at 4°C for 30 minutes. Antibodies were purchased from Biolegend unless otherwise stated. Samples were acquired using the Attune flow cytometer (Invitrogen), and analyses were performed using FlowJo 10.7.1 software.

### Human supplemented media.

Human supplemented media were made with RPMI 1640 media with glutamine, 1× nonessential amino acids, 1 mM sodium pyruvate, 0.4X MEM vitamin solution (Gibco), 92 μM 2-mercaptoethanol, 10 μM ciprofloxacin, and 10% Human AB serum (GemCell, Gemini BioProducts).

### Data and code availability.

Codes were implemented using Python 3.8 and R, which were uploaded to Github (44). Links to the public databases and TCR clustering algorithms are also provided. Donor TCR sequencing data are available upon reasonable request.

### Statistics.

Wilcoxon matched-pairs signed-rank 2-tailed test was performed for matched sample comparisons. Spearman’s rank correlation (2-sided) was used to test for significance and *r* value (correlation coefficient). Two-sided 1-way ANOVA with Tukey’s multiple comparisons test was used to test for significance for matched sample comparisons. For V gene enrichment, the χ^2^ test was performed in R. All other statistical analysis was performed using GraphPad Prism 6 software. *P* = 0.05 was used as our threshold for significance.

### Study approval.

The study design and research protocol were approved by the Johns Hopkins School of Medicine Institutional Review Board. Written informed consent was obtained from all participating donors before their inclusion in this study. Convalescent samples were also obtained from the MITZVA study (24). The study design and research protocol for this study were approved by the IntegReview Institutional Review Board (Austin, Texas, USA). Characteristics of all donors in the study are provided in Tables 1 and 2.

## Author contributions

WC, HW, TK, SFS, JGB, JPS, and AI designed and discussed the project. WC, HW, and TK conducted the investigation, acquired data, analyzed data, and wrote the manuscript. SFS, JGB, SL, HW, MLZ, and NKL helped process samples. HW performed computational analysis for cross-reactive TCR sequences. AZR and IZ participated in the coordination of the study and enrollment of MITZVA blood donors. JJH and RES helped with blood draws. JPS supervised the project. All authors read and approved the final manuscript.

## Supplementary Material

Supplemental data

## Figures and Tables

**Table 1:**
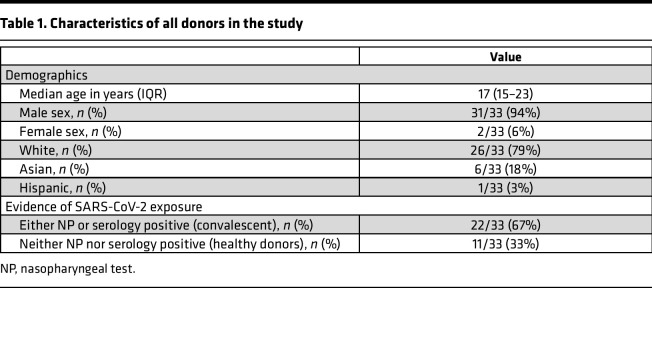
Characteristics of all donors in the study

**Table 2:**
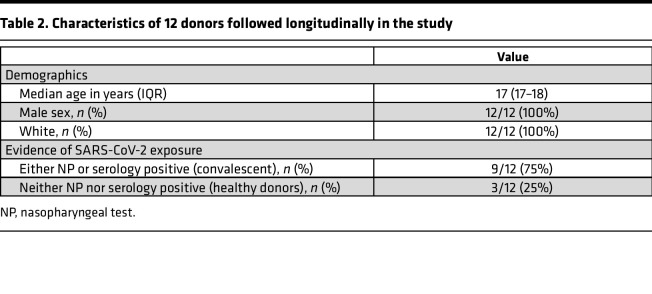
Characteristics of 12 donors followed longitudinally in the study

## Version 1 09/22/2022

Electronic publication
