# Supplementary material for: Cross-reactivity of SARS-CoV-2– and influenza A–specific T cells in individuals exposed to SARS-CoV-2
Source: JCI Insight. 2022 Sep 22;7(18):e158308. doi: 10.1172/jci.insight.158308 (PMC9675569; doi:10.1172/jci.insight.158308)
Supplement: Supplemental data [file jciinsight-7-158308-s125.pdf]

Supplemental Table 1. Peptide names, sequences, protein regions and antigens used in the study.

Peptide Name	Peptide sequence	Protein region	Antigen
YLQ*	YLQPRTFLL	S	SARS-CoV-2
LLL*	LLLDRLNQL	N	SARS-CoV-2
KLW*	KLWAQCVQL	ORF1ab	SARS-CoV-2
ALW*	ALWEIQQVV	ORF1ab	SARS-CoV-2
YLF*	YLFDESGEFKL	ORF1ab	SARS-CoV-2
LLY*	LLYDANYFL	ORF3a	SARS-CoV-2
M1	GILGFVFTL	M	Influenza A
MART1	ELAGIGILTV	-	Melanoma antigen recognized by T cells 1

S= Spike protein, ORF=Open Reading Frame, N=Nucleocapsid protein, M= Matrix protein

*Reference epitopes from Ferretti, Andrew P., et al. (2020)

Supplemental Table 2. iSMART clustering result of TCRs specific to M1 and immunodominant SARS-CoV-2 epitopes used in the qPCR study.

Epitope	Unique ImmuneCODE Entries	M1 entries with exact CDR3β match	M1 entries with similar CDR3β (iSMART)
LLL	1073	6	203
KLW	769	0	140
YLQ	1448	0	287
ALW	5	0	0
YLF	0	0	0
LLY	1130	3	543

Supplemental Table 3. Pre-designed primers

Target gene	Assay ID	Dye
IFNG	Hs00989291_m1	FAM/MGB
HPRT1	Hs02800695_m1	VIC/MGB_PL
CD8	Hs00233520_m1	ABY/QSY

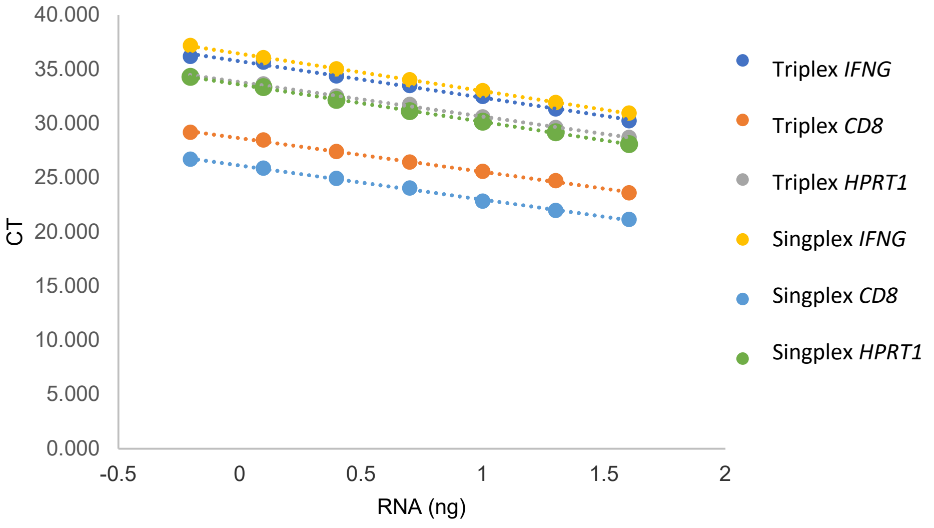

Standard Curve	Slope	R^2	E
Singlex *IFNG*	-3.4427	0.9998	95
Triplex *IFNG*	-3.3599	0.9949	98
Singlex *HPRT1*	-3.1892	0.9975	106
Triplex *HPRT1*	-3.4307	0.9994	96
Singlex *CD8*	-3.0977	0.9980	110
Triplex *CD8*	-3.1456	0.9982	108

Supplemental Figure 1. Evaluation of RT-qPCR primer efficiencies. The amplification efficiency for each primer threshold cycle (Ct) and the logarithm of the initial RNA concentrations were plotted to calculate the slope (S) of each primer pair. Standard curves were generated from eight dilution points for each primer pair. RT-qPCR reactions for each sample were run in triplicate, with standard deviations <0.4. The efficiency of all designed primer pairs ranged from 80% to 110%, which is considered the optimal efficiency value

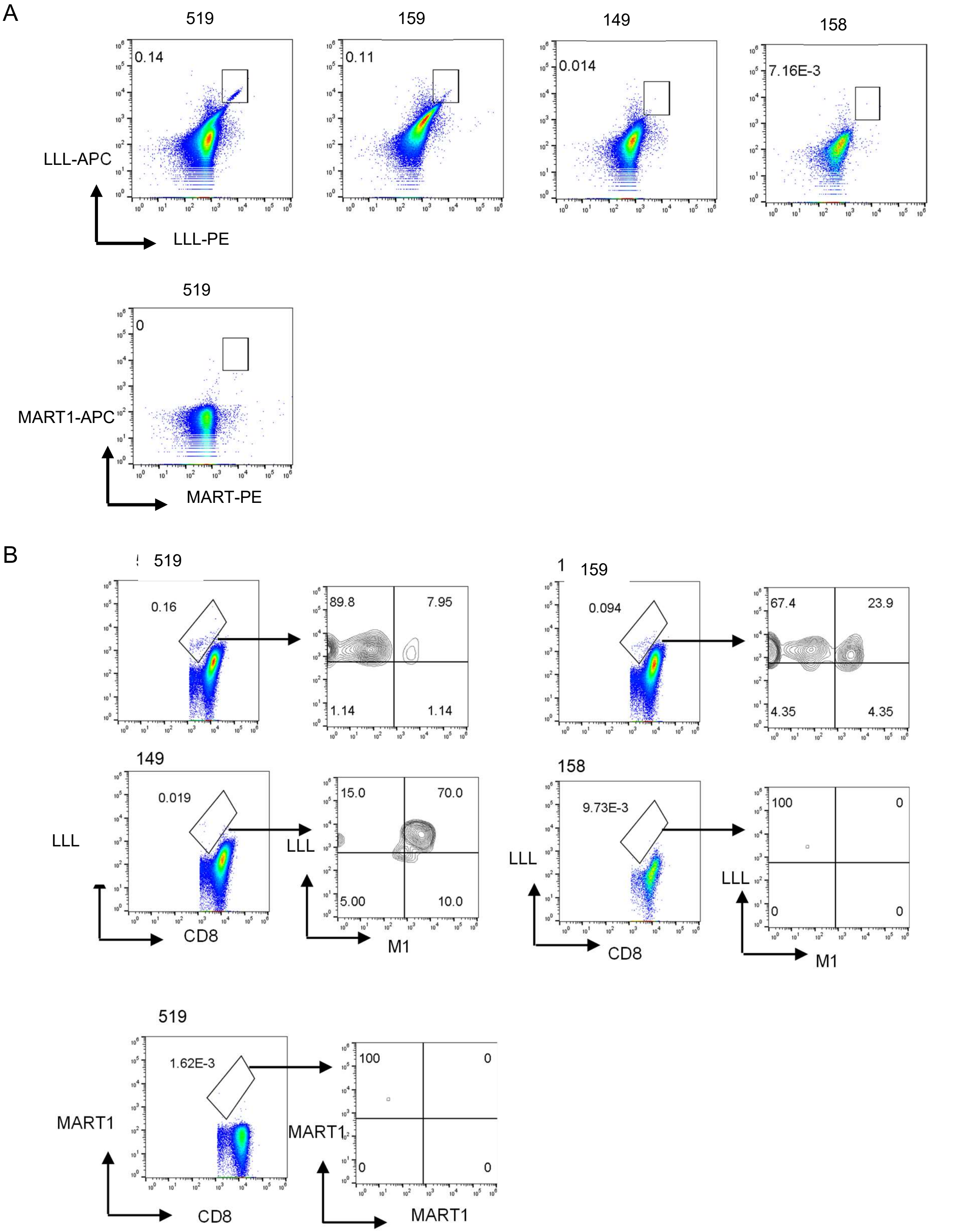

Supplemental Figure 2 Evidence of LLL/M1 CD8 T cell cross reactivity in PBMCs of COVID-19 convalescent individuals by tetramer co-staining directly ex vivo on expanded with mixed LLL and M1 for14 days (D14).

(A) Double staining of LLL-APC and LLL-PE demonstrated the population of LLL after expansion 14 days with mixed LLL and M1 aAPC. **(B)** Double staining of LLL-APC and M1-PE demonstrated the M1 population in subset of LLL population after expansion 14 days with mixed LLL and M1 aAPC. MART1 is used as a non-cognate tetramer.

149 D14 expanded with LLL and M1

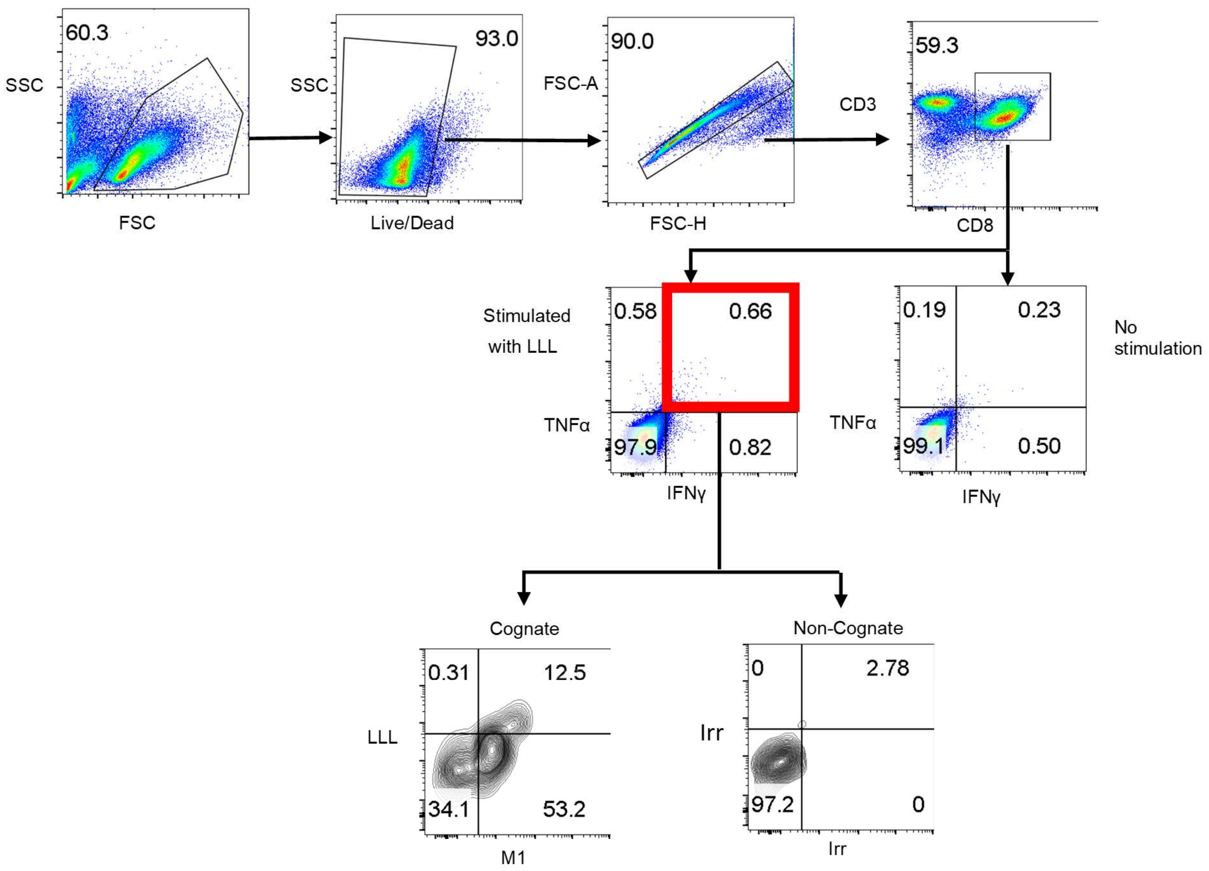

Supplemental Figure 3. Gating strategy for co-staining tetramers and intracellular cytokine staining (ICS) after 14 days PBMC expansion with LLL and M1 aAPC and stimulated with LLL on day 14. Negative tetramer gate is based on non-cognate staining.

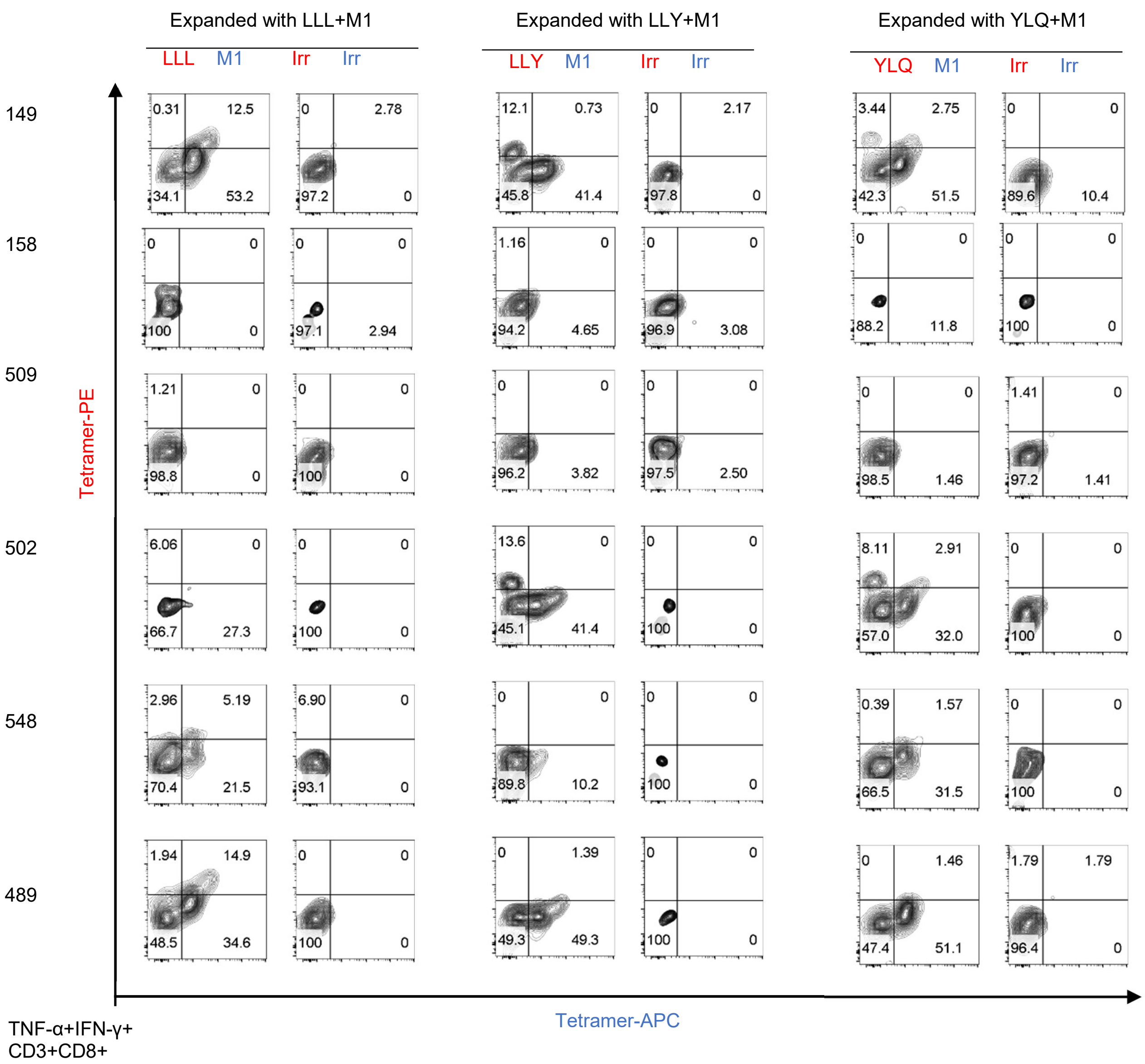

Supplemental Figure 4. Flow plots of TNF-α+IFN-γ+ CD3+CD8+ T cell populations following a 14-day expansion of PBMC from COVID-19 convalescent individuals with mixed LLL and M1. Flow plots illustrate co-staining of cytokine expressing CD8 T cells with both SARS-CoV2 tetramer (LLL, LLY and YLQ) and M1 tetramer vs irrelevant (Irr) tetramer following a brief, 6-hour stimulation with LLL aAPC.

PBMC from 9 COVID-19 convalescent donors were cultured for 14 days in the presence of mixed 1) LLL and M1 2) LLY and M1 3) YLQ and M1 aAPC before being stimulated with LLL aAPC versus no stimulation for 6 hours. The proportion of cytokine expressing CD8 T cells is denoted as % of the TNF-α+IFN-γ+ CD3+CD8+ population.

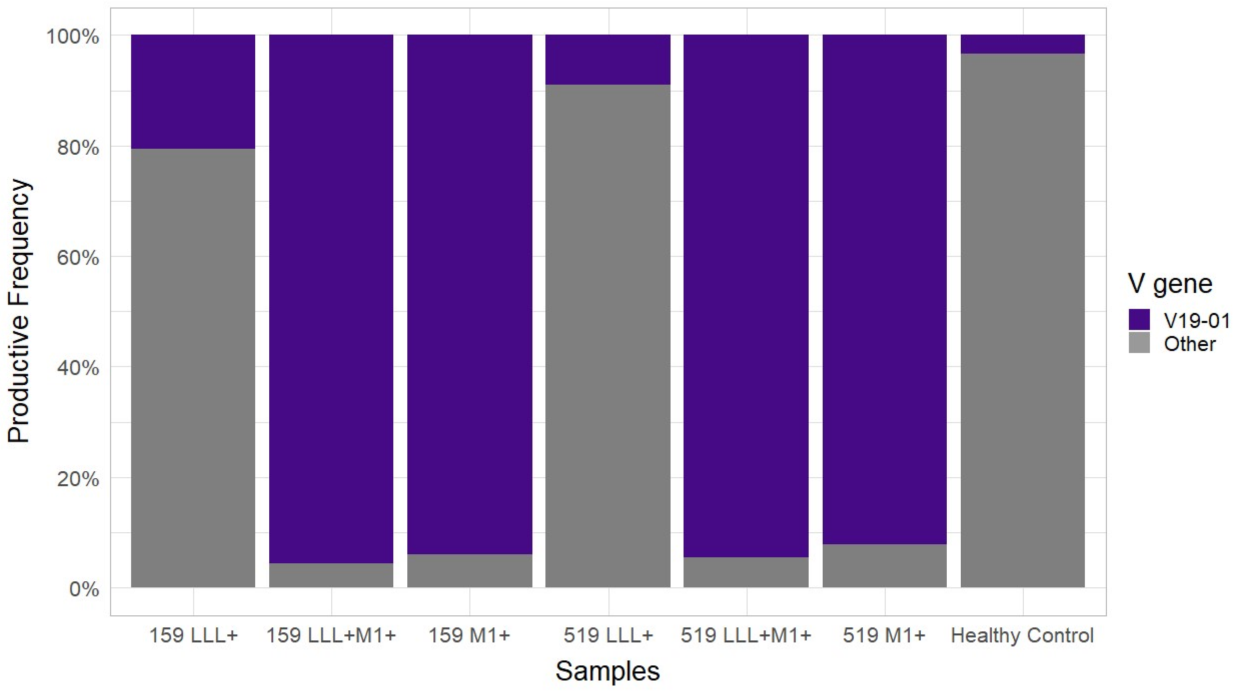

Supplemental Figure 5. TCRBV19-01 gene usage in all samples.
